# Nontoxic electron collimators

**DOI:** 10.1002/acm2.13398

**Published:** 2021-09-04

**Authors:** Dylan Yamabe Breitkreutz, Lawrie Skinner, Stephanie Lo, Amy Yu

**Affiliations:** ^1^ Department of Radiation Oncology Stanford University Stanford California USA

**Keywords:** 3D printing, electron therapy, skin collimation

## Abstract

**Purpose:**

The goal of this work was to develop and test nontoxic electron collimation technologies for clinical use.

**Methods:**

Two novel technologies were investigated: tungsten‐silicone composite and 3D printed electron cutouts. Transmission, dose uniformity, and profiles were measured for the tungsten‐silicone. Surface dose, relative dose output, and field size were measured for the 3D printed cutouts and compared with the standard cerrobend cutouts in current clinical use. Quality assurance tests including mass measurements, Megavoltage (MV) imaging, and drop testing were developed for the 3D printed cutouts as a guide to safe clinical implementation.

**Results:**

Dose profiles of the flexible tungsten‐silicone skin shields had an 80–20 penumbra values of 2–3 mm compared to 7–8 mm for cerrobend. In MV transmission image measurements of the tungsten‐silicone, 80% of the pixels had a transmission value within 2% of the mean. An ∼90% reduction in electron intensity was measured for 6 MeV and a 6.4 mm thickness of tungsten‐silicone and 12.7 mm thickness for 16 MeV. The maximum difference in 3D printed cutout versus cerrobend output, surface dose, and full width at half‐maximum (FWHM) was 1.7%, 1.2%, and 1.5%, respectively, for the 10 cm × 10 cm cutouts.

**Conclusions:**

Both flexible tungsten‐silicone and 3D printed cutouts were found to be feasible for clinical use. The flexible tungsten‐silicone was of adequate density, flexibility, and uniformity to serve as skin shields for electron therapy. The 3D printed cutouts were dosimetrically equivalent to standard cerrobend cutouts and were robust enough for handling in the clinical environment.

## 1 INTRODUCTION

Currently, collimation of clinical electron beams is commonly performed by shaping low melting point alloys (typically Bi–Zn–Pb–Cd), known as cerrobend, to the target shape. Although contact with the final solid form is relatively safe, the process involves maintaining a pot of molten alloy and venting the toxic lead and cadmium fumes outside the building and into the local environment. Where higher precision treatments are needed, skin collimation devices are often used. Due to its combination of density and ductility, lead is the most common skin collimation material. The patient skin contact, staff handling, and lead waste are all toxicity hazards. There is no known minimum safe level of lead in the human body, and there is almost no function in the human body, which is not affected by lead toxicity.^1^

Existing efforts toward improving electron radiotherapy range from large complicated and expensive electron multileaf collimator systems^2^, ^3^ to relatively simple variable thickness bolus.^4^, ^5^ Specifically, on static aperture collimation devices, work has been performed using 3D printed molds to improve accuracy of the cerrobend casts, that also allows for gridded beams.^6^ Our prior work^7^ investigated the basic feasibility of 3D printed, tungsten‐filled electron beam collimation apertures. For skin collimation devices, 3D surface scanning has been shown to be useful in aiding their design,^8^ while tungsten‐filled rubber and tungsten‐filled paper have also been shown to effectively collimate electron radiotherapy beams.^9^, ^10^

In the present work, two clinically tested devices are presented for collimation of clinical megavoltage electron beams, which between them, cover a wide range of clinical situations for electron radiation therapy. The first device is a flexible‐tungsten composite material used for both skin collimation and island blocking, which can be used instead of lead. The second method involves 3D printed, tungsten‐filled, electron cutouts (3D‐EC), which replace the low melting point alloy insert with a 3D printed shell filled with tungsten spheres with 1–2 mm diameter. The purpose of this work is to describe a practical method for the use of these devices in the clinic.

## 2 METHODS

### 2.1 Flexible tungsten‐silicone skin collimators

**FIGURE 1:**
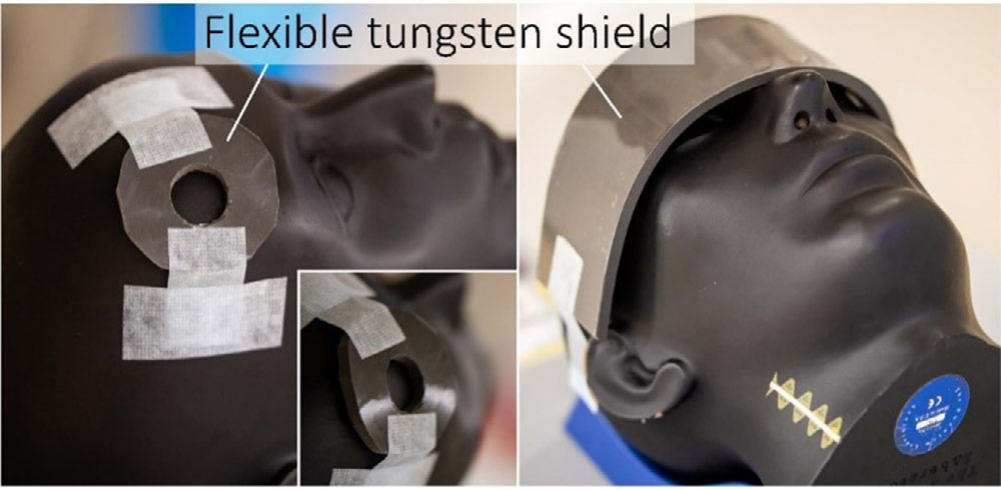
Flexible‐tungsten sheet 6 mm thick and ∼7 g/cm^3^ density. The silicone‐tungsten composite is sufficiently flexible to conform to patient surfaces

Composite silicone sheets containing tungsten powder were purchased from Lancs Industries (Kirkland WA, USA). The nominal thickness was ¼ in (6.35 mm) at a nominal density of 250 lb/in^3^ (6.92 g/cm^3^). Inspection with calipers and weighing scales confirmed these values within measurement uncertainties of ±0.2 mm and ±0.5 g/cm^3^. The material was found to be flexible with radius of curvature of 4 cm and greater easily achievable with minimal effort (Figure 1). On one side, a thin layer of silicone sealant was added to help seal in any tungsten powder that my come loose. This was not necessary on the other surface as this already had a thin clear silicone sealant top layer. The material is easily cut with scissors or small blades, but hot wire cutting is not expected to work due to the high thermal stability of silicone.

Transmitted dose was measured for single‐ and double‐layered sheets for 6, 9, 12, 16, and 20 MeV electron energies on a Varian Truebeam (Varian Medical Systems, Palo Alto, CA, USA), where the energies were matched to the Varian reference dataset. An exradin A10 parallel plate ion chamber (Standard Imaging, Middleton, WI, USA) was placed at the surface and at 10 cm depth, of a solid water phantom with a constant source‐surface distance (SSD) of 100 cm. The ratio of the measured charge with and without the 6 mm flexible‐tungsten shield placed on top of the phantom was obtained for the two depths.

Once central axis attenuation was established, the uniformity of the flexible‐tungsten collimators was investigated via imaging with a 6 MV FFF beam. Images were taken with and without the flexible‐tungsten sheet placed on the detector panel cover. The ratio of open to attenuated fields was then taken to determine the attenuation map. The FFF beam was used as the spectrum is relatively constant with off‐axis distance.^11^ The intensity values in the ratio image were then logged ($\ln\;(I/I_{0})=\;-\mu t$). A histogram of these μt values was then compiled to evaluate the constancy of the radiological thickness of the flexible‐tungsten sheet.

In‐phantom dose profiles were measured using radiochromic film for a 9 MeV electron beam. To match clinical situations, the phantom was placed at an SSD of 105 cm and the film was placed at a depth of 0.5 cm. A total of 400 MU deliveries were then made with and without the flexible‐tungsten circular aperture placed on the phantom surface. For the measurements without a flexible‐tungsten circle, a 2.2 cm diameter circular cerrobend aperture was used to create a small field size. For the flexible‐tungsten exposures, a wider 3.9 cm circular cerrobend circle was used, with 2.0 cm or 2.6 cm diameter flexible‐tungsten circular apertures placed on the phantom surface. All field sizes are their projected size, as shown by the light field, at the 105 cm SSD phantom surface.

To understand the excess backscattered radiation from the flexible‐tungsten material, measurements were made using a 1 mm × 1 mm W2 scintillator detector (Standard Imaging) with 1 cm superflab bolus on top and varying solid water thickness between the detector (upstream) and flexible‐tungsten sheet (downstream). A constant SSD of 104 cm was used. This setup represents a common clinical use case of 1 cm bolus and 105 cm to the skin. The ratio of dose with and without the flexible‐tungsten sheet in place was then calculated for nominal electron energies of 6, 9, and 12 MeV on a Varian Truebeam matched to the standard Varian reference data.

These tests were chosen as they establish the attenuation, uniformity, and field edge profiles provided by this flexible‐tungsten material as a skin collimator for small electron apertures. For larger field sizes, skin collimation is not typically necessary, and custom 3D printed applicator inserts can be used.

### 2.2 3D printed electron cutouts

#### 2.2.1 Design and manufacture

**FIGURE 2:**
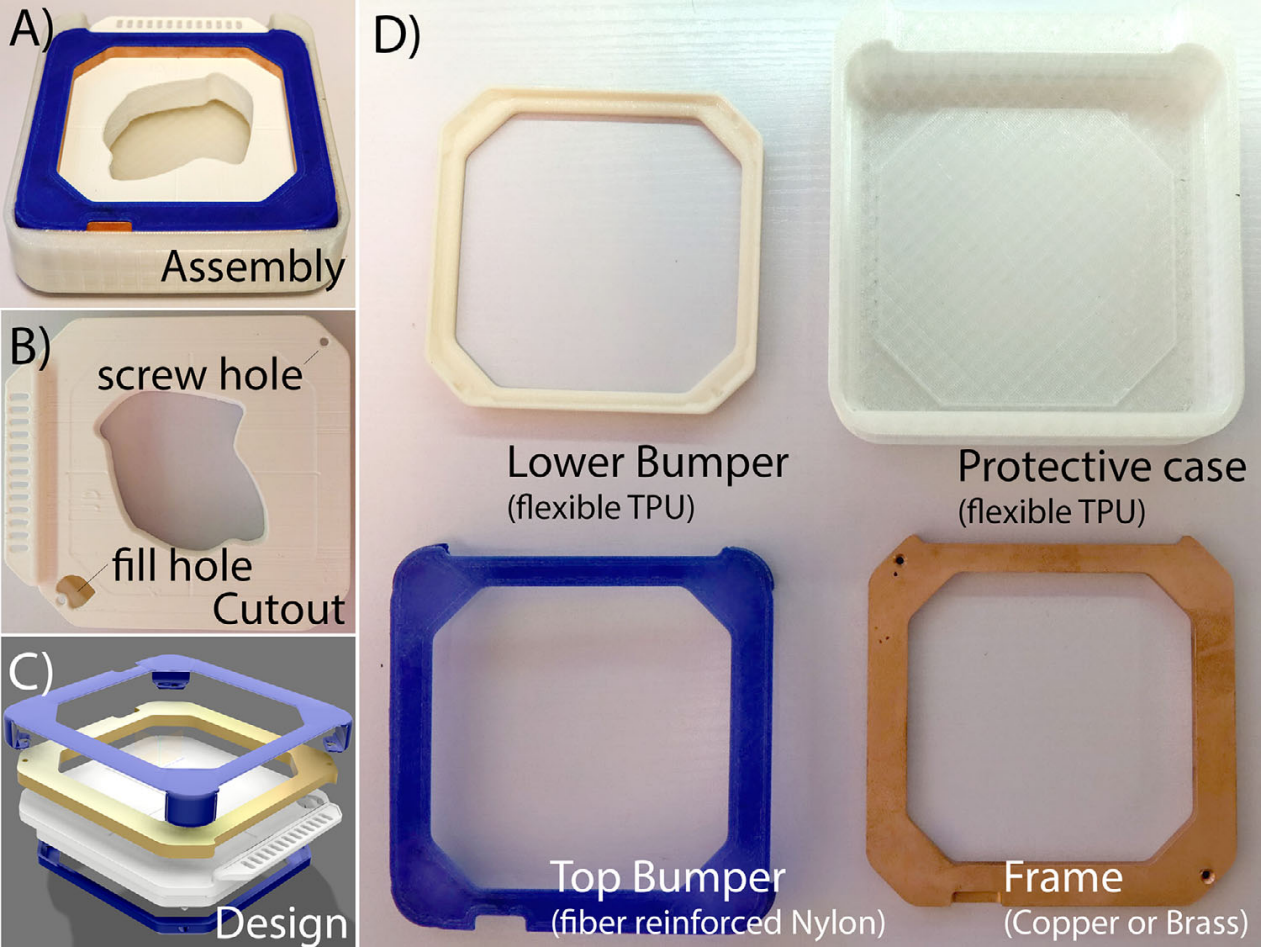
3D printed components of the electron cutouts: (A) assembled part in protective case, (B) main 3D printed part., (C) assembly diagram of the components, and (D) individual components: the two bumpers and case are for impact protection in case the cutout is dropped, the copper frame adds both strength and shielding of the edges in case of incomplete BB filling

The 3D printed cutout consisted of five components: the rigid plastic cutout, a copper frame, a flexible lid, a soft protective case, and tungsten ball bearings (BBs; Figure 2).

The design took this form to enable it to withstand a drop from 1 m height onto a hard floor without a catastrophic release of BBs into the room. For speed and ease of printing, the cutout was 3D printed using tough PLA plastic, this is the only nonreusable, patient‐specific component. The frame was computer numerical control (CNC) machined from brass or copper (8.5–9.0 g/cm^3^). The purpose of the frame is to provide shielding of the field edges even when the cutout is slightly underfilled and the gantry is rotated from zero. The lid is printed from carbon fiber reinforced nylon and provides protection to the corners of the cutout component in the event it is dropped. The final part is the protective case, which is 3D printed from a flexible thermoplastic polyurethane (TPU) plastic.

All the template parts were designed in Fusion 360 (Autodesk, San Rafael, CA, USA). Template cutouts were created, which fit the 6 cm × 6 cm and 10 cm × 10 cm electron cones of Varian linacs. Electron apertures were then designed in the Eclipse treatment planning system (Varian Medical Systems) as they would be for any CT‐based treatment plan. The dimensions of the electron aperture was exported from Eclipse and imported into Tinkercad to produce the .stl file of the electron cutout to be printed. The .stl file was exported to Cura (Ultimaker B.V., Utrecht, The Netherlands) for processing prior to transfer to an Ultimaker S5 (Ultimaker B.V.) 3D printer for printing.

After printing, the 3D printed cutout was filled with tungsten BBs with diameters between 1.5 and 2 mm. To reduce air gaps, the cutout was rotated and shaken during the filling process. The separate components of the 3D printed cutout were then assembled. Underfilling is also investigated and discussed in Sections 3.2.1 and 4.2. The 10 cm × 10 cm electron cutout aperture was a 7 cm diameter circle, whereas the 6 cm × 6 cm electron cutout aperture was a 5 cm diameter circle.

#### 2.2.2 3D printed cutout verification and quality assurance

Three quality assurance tasks of the 3D printed cutouts were performed: (1) weight measurement, (2) MV portal imaging, and (3) drop testing. The first two are suggested for each patient‐specific cutout. The drop testing is (usually) destructive and was used in this work to evolve the design toward a more robust solution.

The fill fraction of the tungsten BB filling can be calculated with a weight measurement and the internal volume of the cutout. These can be used to calculate a target weight to ensure the 3D printed cutout has been adequately filled. The density of the tungsten BB filled volume is less than that of solid tungsten due to air gaps between the BBs. The average density of the air and tungsten volume, $\rho_{cal}$, was determined by 3D printing a simple rectangular box with easily measurable dimensions, filling it with tungsten BBs and measuring the mass of the BBs when the box was fully filled. This average density of tungsten BBs and air (packing fraction) should be consistent among all cutouts when fully filled. Then the QA check for adequate filling is

(1) $$\left(m_{f}-m_{0}\right)\geq \rho_{\mathrm{cal}}\cdot \left(V_{\mathrm{des}}+C\right)\cdot \;tol,$$

where $m_{f}$, $m_{0}$ are the measured masses of the fully filled and empty cutouts, respectively. tol is the lower limit on the acceptable fill fraction. This tolerance should be determined through commissioning tests (suggested range 0.97–1.0). $V_{\mathrm{des}}$ is the internal volume from the design file and *C* is a constant offset correction to the internal volume.

The added steps of first measuring $\rho_{\mathrm{cal}}$ in a box of known internal volume, and then applying an offset correction, *C*, to the design volume, *V*_des_, was used as the exact internal dimensions of the 3D printed cutouts have up to a few percentage of offset in the internal volume compared to the design. This offset depends on slicing settings. The correction, *C*, to the internal volume of the patient‐specific designs, $V_{\mathrm{des}}$ was determined from a calibration set of weight measurements, of small and large apertures, and is treated as a fixed constant for each applicator size. Specifically:

(2) $$C\;=\frac{\left(m_{f}-m_{0}\right)}{\rho_{\mathrm{cal}}}\;-V_{\mathrm{des}}$$

on set of *m_f_*, *m*_0_ measurements, and the designed internal volumes, $V_{\mathrm{des}}$ of those parts. For example, if one plots the theoretical *V*_des_ against the measured mass of BB's (*m_f_* − *m*_0_) for a few cutout sizes, then the intercept of a linear fit will give the value *C*. Also optimally, one may use the slope as an effective $\rho_{\mathrm{cal}}$. If this offset correction is not made, that is, the fit is forced to have zero intercept, the predicted mass was found to be insufficiently accurate.

The second QA check is to take an MV portal image through the filled cutout at the treatment gantry and collimator angles, to confirm that the BB's sufficiently cover the field edges, without gaps in the highest part of the cutout.

Lastly, the drop test, involved dropping a fully filled and assembled 3D printed cutout from a height of 1 m onto a hard stone surface. A height of 1 m was chosen as it is close to the height that the cutouts are normally transported.

#### 2.2.3 Dosimetry measurements

To ensure that there were no substantial dosimetric differences between the 3D printed and cerrobend cutouts, several different dosimetric tests were performed.

Relative dose measurements were performed with solid water and an ionization chamber to compare the output of the 3D printed and cerrobend cutouts. A Varian Truebeam STx (Varian Medical Systems) was used for all measurements. The experimental setup consisted of 10 cm of solid water backscatter, a solid water slab within which a pinpoint chamber (PTW, Freiburg, Germany) was inserted and various solid water slabs to adjust the depth of the chamber. The source‐to‐surface distance was set to 100 cm. A Max4000+ (Standard Imaging) electrometer was used to read the charge collected by the ionization chamber. Four hundred megaunits were delivered with electron beam energies of 6, 9, 12, 16, and 20 MeV at depths of 1.5, 2.2, 3, 4, and 5 cm (approximate values of *d*_ref_), respectively, for both the 6 cm × 6 cm and 10 cm × 10 cm cerrobend and 3D printed cutouts.

Surface dose measurements were performed using solid water and a parallel plate ionization chamber. Ten centimeters of solid water was used for backscatter and a 2 cm slab of solid water with a parallel plate insert was placed on top. 0, 0.2 and 0.5 cm of solid water was placed on top of the chamber surface for different measurements. One hundred megaunits of 6 and 20 MeV electrons were delivered with the 7 cm diameter 10 cm × 10 cm 3D printed cutout and the 7 cm cerrobend cutout.

To evaluate the field size of the 3D printed cutouts, an IC Profiler (Sun Nuclear Corporation, Melbourne, FL, USA) measurement device was used to measure dose profiles for each electron energy. Various amounts of solid water was placed on the IC Profiler depending on the electron energy being measured. The chosen depths were those used clinically for monthly electron QA measurements with the IC Profiler. Values of field size, defined as the full width at half‐maximum (FWHM) of the measured profile were reported.

To ensure that the tungsten BB filling adequately attenuated the electron beam, EBT3 Gafchromic film (Ashland Global, Wilmington, DE, USA) was used to evaluate leakage. Gafchromic film was placed on top of 10 cm of solid water with 0.6 cm of solid water on top with an SSD of 100 cm. The collimator was set to 315° and the gantry was set to 90°. Eight hundred megaunits of 16 MeV electrons were delivered using the 10 cm × 10 cm 3D printed cutout both when filled to 99% capacity by weight. The film were then inspected for indications of leakage through the BB filling.

## 3 RESULTS

### 3.1 Flexible tungsten filled silicone skin collimators

**FIGURE 3:**
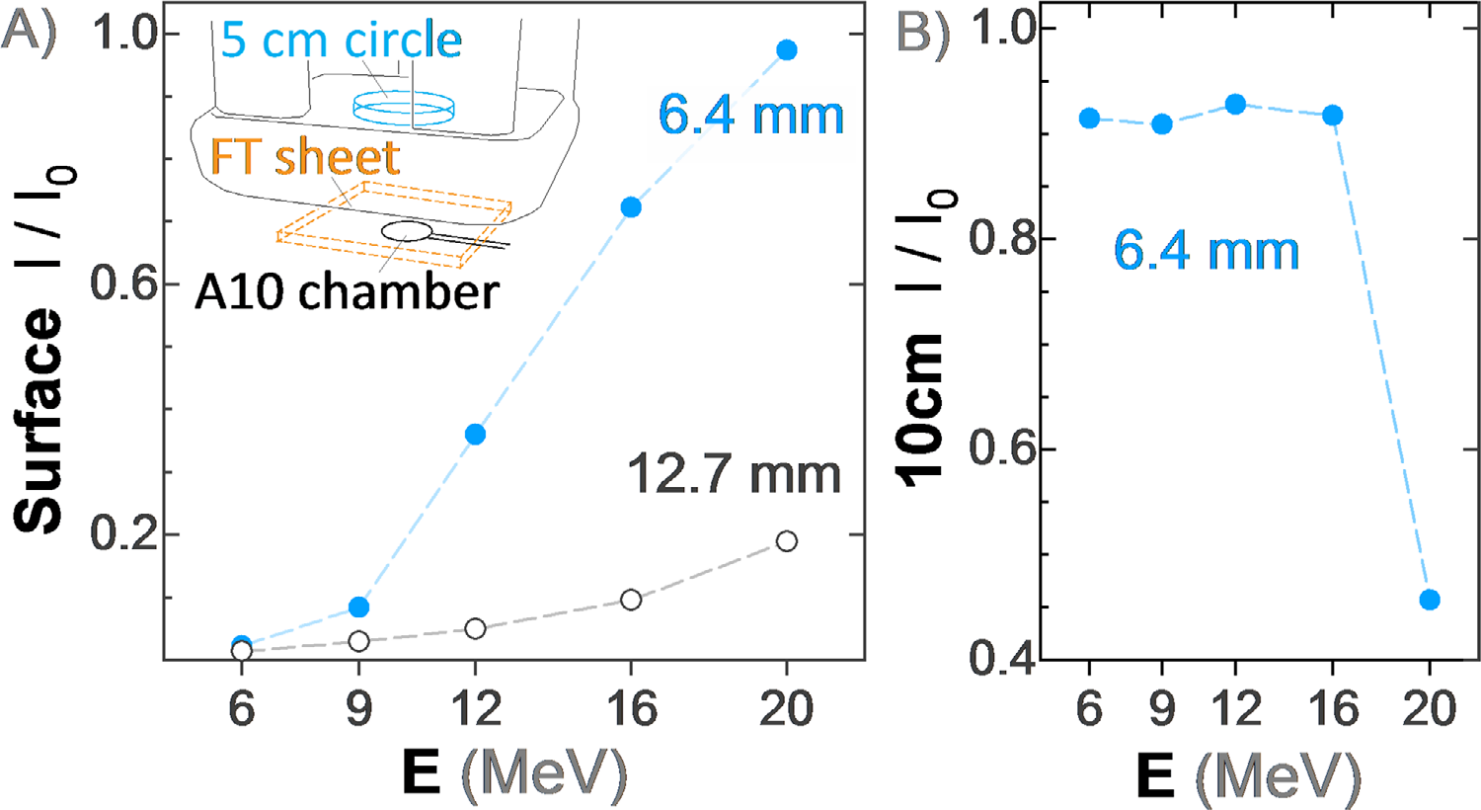
(A) Single‐layered (6.4 mm thick) and double‐layered (12.7 mm) flexible‐tungsten skin collimators with a 5 cm cerrobend circle in a 6 cm × 6 cm applicator, measured with an A10 parallel plate ion chamber at SAD 100 cm. Transmitted dose, *I*/*I*_0_, is the ratio of measurements with and without the flexible‐tungsten layers. (B) The same but at a measurement chamber depth of 10 cm in a solid water phantom. Note that in (b) *I*/*I*_0 _< 1 indicates that Bremsstrahlung dose is reduced by placement of the flexible‐tungsten skin collimator (i.e., it attenuates more X‐rays than it creates)

Transmission measurements through either a single or double layer of flexible‐tungsten sheets are shown in Figure 3. Measurements were made at the phantom surface, immediately beneath the flexible‐tungsten sheet (Figure 3a) as well as at 10 cm below the flexible‐tungsten sheet in a solid water phantom (Figure 3b).

**FIGURE 4:**
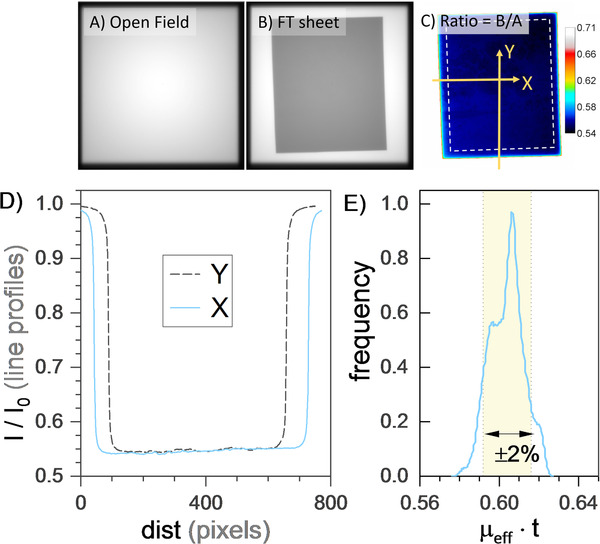
(A,B) Six megavolt FFF images and (C) their ratio. (D) A profile of the ratio and (E) a histogram of the ratio image after the image scale ”logged” to provide ln(*I*/*I*_0_), which is equal to (μ/ρ)⋅ρ⋅t. The histogram is a measure of homogeneity of the material's radiological thickness

The uniformity of the attenuation provided by the flexible‐tungsten sheets is given in Figure 4, which plots the range of effective radiological thickness in a sample sheet. From this histogram, 80% of the area was within 2% of the mean. This measurement is suggested as a commissioning test of the homogeneity of each sheet before implementing such devices.

**FIGURE 5:**
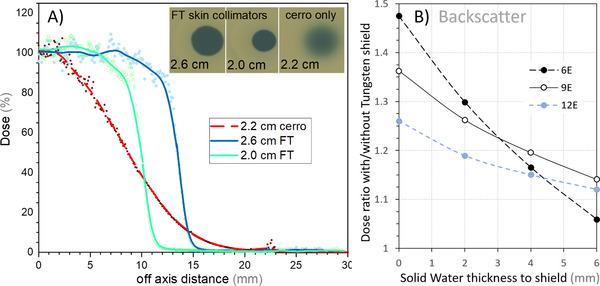
(A) Radiochromic film dose profiles at 0.5 cm depth for small circular electron fields on a solid water phantom placed at 105 cm source‐surface distance (SSD) and irradiated with a 9 MeV electron beam. Fields were defined by either the cerrobend aperture in the Varian electron applicator, or by flexible‐tungsten “donut” apertures placed on the phantom surface (see the inset in Figure 1). The circle diameters (2.0, 2.6, 2.2 cm) are the sizes as shown by the light field on the phantom surface. (B) Backscattered dose measurements upstream of the flexible‐tungsten sheet. Measurements are made with 1 cm bolus on top of a W2 detector with a variable thickness below, between the detector flexible‐tungsten sheet

Figure 5a shows measured dose profiles for the radiochromic film method described in Section 2.1, in which measurements with and without a flexible‐tungsten skin collimator are compared at a typical treatment SSD of 105 cm. Figure 5b shows the results of measurements of backscattered dose upstream of the flexible‐tungsten sheet. The dose profiles with the flexible‐tungsten surface collimator have 80–20 penumbra widths of 2–3 mm at the 0.5 cm measurement depth, compared to 7–8 mm for the cerrobend defined field without surface collimation.

### 3.2 3D printed electron cutouts

#### 3.2.1 3D printed cutout verification and quality assurance

**FIGURE 6:**
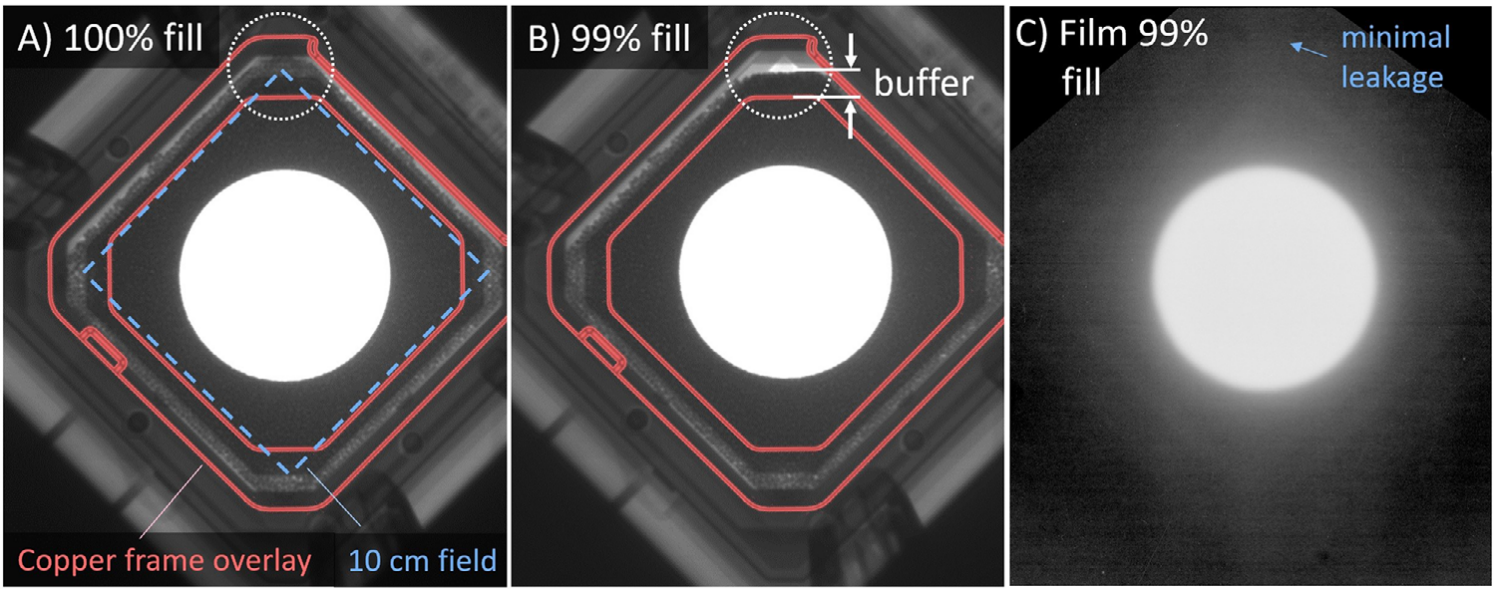
(A,B) Six megavolt port images at gantry angle 90° of the 7 cm diameter 10 cm × 10 cm cutout when fully filled (a) and when underfilled by 1% (B). The 3D printed cutout was filled to 99% by weight. In all cases the collimator angle was 45° and the gantry was at 90° to create the worst case for gaps in the BB layer from gravity. No significant leakage was observed in the radiochromic film. (C) Photograph of EBT3 Gafchromic film irradiated at 0.6 cm depth in solid water and 5000 MU of 20 MeV electrons to ensure there is no leakage

The weight of the tungsten BBs filling the rectangular box was 1185.3 g. The measured volume of the box was 118.1 cm^3^, yielding a BB density $\rho_{cal}=\;10.04\;\pm \;0.05$ g/cm^3^. The standard deviation of the measured mass of the BB filling was 3.4 and 3.9 g. The calculated densities were 9.85 and 10.29 g/cm^3^. The image of the Gafchromic film irradiated with 20 MeV electrons is presented in Figure 6. No signs of leakage through the cutout were observed.

MV images of the 7 cm diameter 10 cm × 10 cm 3D printed cutout is shown in Figure 6a,b. The cutout was filled fully and to 99% by weight, that is, deliberately 1% underfilled. Images were taken with the gantry at 90° and the collimator at 45°. The brass frame is observed to cover the region of underfill (left corner of second image), which is its intended purpose. Minimal leakage was observed in the Gafchromic film as well. After dropping the final design from approximately 1 m in height, the 3D printed cutout held together and did not lose any of the tungsten BB filling.

#### 3.2.2 Dosimetry

The relative output dosimetry measurements for the 5 and 7 cm diameter circular cutouts are given in Table 1. For the 7 cm circle in the 10 cm × 10 cm applicator, all output differences were less than 1% between the 3D printed and cerrobend cutouts. The largest difference was 0.7% for the 6 MeV and the smallest difference was 0.2% for the 16 MeV. For the 5 cm circle, the largest difference of 2.3% was seen for 6 MeV and the smallest difference was 0.6% for the 20 MeV.

**TABLE 1:** Relative output measurements for the 7 cm diameter circular 3D printed and cerrobend cutouts in a 10 cm × 10 cm applicator and 5 cm diameter circles in a 6 cm × 6 cm applicator

		Central axis output differences relative to cerrobend cutout (%)		
Energy (MeV)	Measured depth (cm)	7 cm circle	Patient cutout	5 cm circle
**6**	1.5	0.7	1.7	2.3
**9**	2.2	0.2	1.6	1.8
**12**	3	0.2	1.5	1.7
**16**	4	0.2	1.4	0.6
**20**	5	0.4	1.1	0.6

Surface dose measurements are given in Table 2. The largest difference between the 3D printed and cerrobend cutouts was 1.16% observed at a depth of 0.5 cm for 6 MeV. The smallest difference was 0.31% for 20 MeV at a depth of 0.5 cm.

**TABLE 2:** Surface dose measurements for the 7 cm diameter 10 cm × 10 cm 3D printed cutout and 7 cm cerrobend cutout

	6 MeV CAX	20 MeV CAX
Depth (cm)	Difference (%)	Difference (%)
0	0.9	1.0
0.2	0.8	0.6
0.5	1.2	0.3

*Note*: Measured with a pinpoint micro‐ion chamber.

Measurements of the FWHM for the 5 and 7 cm diameter circles are given in Table 3. For the FWHM for the 7 cm diameter, the largest difference between the 3D printed and the cerrobend cutouts was 1.5% for 6 MeV and the smallest difference was 0.5% for the 20 MeV. For the FWHM for the 5 cm diameter, 6 cm × 6 cm 3D printed and cerrobend cutout, the largest difference was 0.88% for the 20, 6, and 9 MeV measurements matched exactly to the precision of the analysis software.

**TABLE 3:** Measured full width at half‐maximum (FWHM) values for the 5 cm diameter 6 cm × 6 cm 3D and cerrobend cutout

		FWHM difference (%)	
Energy (MeV)	Depth (cm)	7 cm circle	5 cm circle
6	1.1	1.5	0.0
9	1.4	1.3	0.0
12	2.9	0.8	0.5
16	2.5	0.8	0.5
20	3.3	0.5	0.9

## 4 DISCUSSION

### 4.1 Flexible tungsten‐silicone skin collimators

The flexible‐tungsten sheets were found to have a consistent radiological thickness of roughly ±2%. A single 6.4 mm thick sheet was sufficient to attenuate up to 9 MeV (*R*_50_ = 3.6 cm). A double stack of these sheets was sufficient for up to 16 MeV (*R*_50_ = 6.6 cm) but transmits a significant fraction of the 20 MeV electron beam (*R*_50_ = 8.3 cm). Film measurements of a 9 MeV electron field demonstrate that these significantly improve the field edge definition for small electron beams (Figure 5). They are found to be sufficiently flexible for use as skin collimators.

The measurements of backscattered electron dose are most relevant to clinical situations in which tissue is located upstream of the flexible‐tungsten sheet. Such situations may include when the skin collimator is placed under the lip, inside nasal, or oral cavities, between fingers or toes, under the ear lobe, and under the genitalia.

Calculations with the Varian Eclipse treatment planning system using the standard reference beam data and the eMC dose calculation algorithm (v15.6) were found to provide reasonable agreement with the measured transmitted dose when a sheet of thickness 1 cm and density 7 g/cm^3^ (5.8 relative electron density) was used to represent 0.5 cm thick flexible tungsten. that is, the simulated sheet was double the physical thickness. Although not perfect, scaling the thickness is preferred to further scaling the density due to algorithm limitations and because this method reduces the sensitivity of the calculation to contouring inaccuracy.

The dose at 10 cm depth is dominated by Bremsstrahlung X‐rays. This dose is reduced by the presence of the tungsten shield because it attenuates the bremsstrahlung from the head of the linac, more than it creates additional Bremsstrahlung in the tungsten powder. The 20 MeV had approximately 3× higher dose at 10 cm depth than 16 MeV, likely because of some of the 20 MeV electron dose was reaching the 10 cm depth. One can approximate the 0.6 cm and 6.9 g/cm^3^ shield as being equivalent to a 4.2 cm thick water slab.

The flexible‐tungsten skin collimator is approximately 60% the density of lead (6.9 g/cm^3^ vs. 11.3 g/cm^3^). Of high clinical relevance is that these small skin‐collimated fields (<4 cm) have a significant flat region that receives close to the prescription dose, whereas without skin collimation, everywhere apart for the center receives significantly less than the prescription dose, and the area outside the light‐field aperture receives significantly more radiation dose than the skin collimated fields (Figure 5). Hence, for small fields, skin collimation produces improved dosimetry (both lower out‐of‐field dose and higher in‐field dose). Unlike lead sheet, the flexible‐tungsten skin collimators presented here are nontoxic. For larger fields (∼>3 cm), skin collimation is less critical as the area that receives close to the dose is a larger fraction of the light field aperture.

### 4.2 3D printed electron cutouts

Significant cutout design evolution was required since our prior work.^7^ Specifically, issues of robustness, and the potential gaps from underfilled cutouts required design solutions. The copper top plate, soft bumpers, and more evolved designs tested in the present work solve these clinical issues. Specifically, the lower bumper spreads the impact stress through deformation, the rounded designs and integrated lid of the main part make it stronger and avoid stress concentration points. The copper top plate, which is bolted to the 3D printed parts, provides rigidity and strength upon impact, and the nylon top bumper is an optional protective cover to reduce damage to the copper frame.

The output measurements show that the 3D printed cutouts give slightly higher output on central axis than the cerrobend cutouts. This output difference also increases with decreasing aperture size. The precise cause is not determined, but the following was ruled out: out‐of‐field dose was measured to be the same or less for the 3D printed cutouts, Bremsstrahlung dose (dose at depth >10 cm) was less for the 3D printed cutouts, measurements without the plastic top, or without copper bumper were similar to those with the plastic top. Thinner walled 3D prints were found to have output slightly closer to their cerrobend counterparts. From these tests we infer, but have not definitively proven, that the excess output measured for the 3D printed cutouts arises from electrons that are scattered from the walls of the 3D printed aperture. This was also the conclusion in our previous work in which a similar increase in output in the 3D cutouts was observed. In that work, there was a slight increase in the dose 5 cm off‐axis, which supports and also suggests an increase in scatter in the plastic cutouts. Although thinner walled cutouts may solve this, field edge walls thinner than 0.4 mm were found to be insufficiently robust for clinical use.

**FIGURE 7:**
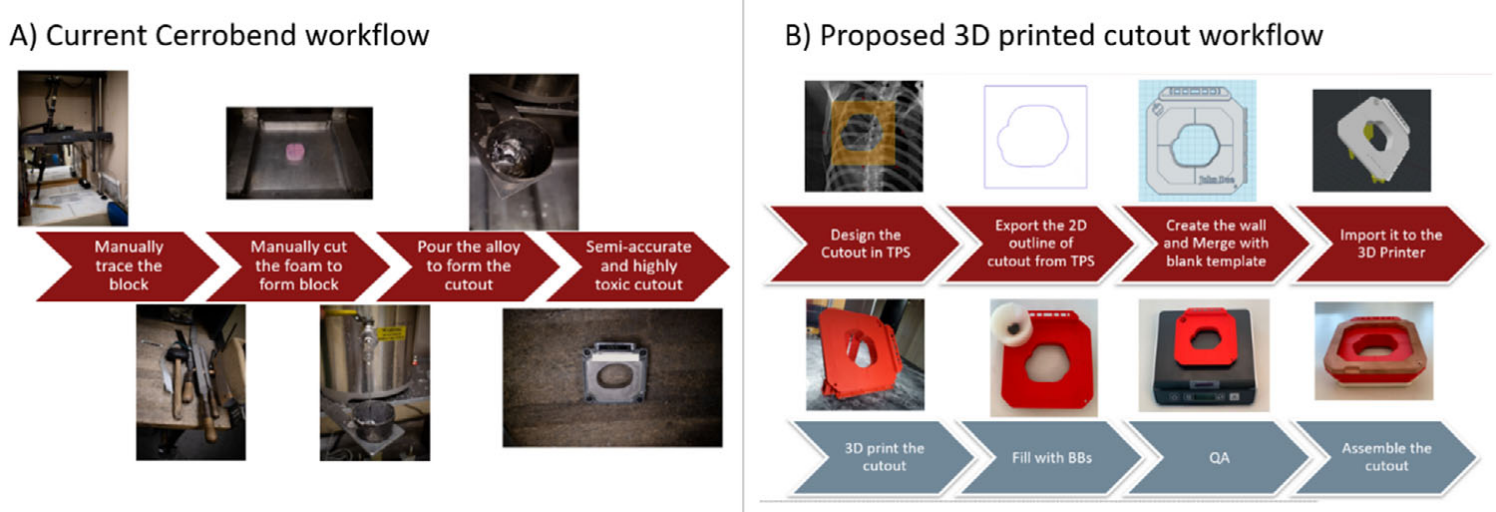
Workflow comparison

The 1% underfilling was investigated in Figure 6 as this is the suggested tolerance for QA weight measurements, such that more than 1% underfilling should be caught at the weight measurement check QA. As each cutout will weigh 500–5000 g, 1% underfilling is a delta of 5–50 g, which is easily measured on commercially available weighing scales with 0.1 g or 1 g precision. For robustness, the 3D printed designs tend to crack along layer line weaknesses and strength is dependent on the material used, and detailed print settings such as print temperature and part cooling. In this work, 220°C was used with matterhackers pro series Tough PLA (www.matterhackers.com) and 25% cooling fan speed, printed on an Ultimaker S5. As strength depends on both design and print settings, it cannot be claimed that a given design is “drop proof,” only that a given print can survive a given impact at least some of the time. A suggested clinical workflow and comparison to the cerrobend workflow is given in Figure 7.

## 5 CONCLUSION

The devices here provide nontoxic, lead‐free, and cerrobend‐free methods to define electron beams through tungsten BB‐filled 3D printed apertures, and flexible composite sheets of silicone tungsten that are suitable for the use as skin collimators. The 3D printed apertures are clinically usable with suitable robustness and dosimetry equivalent to cerrobend apertures. The flexible silicon‐tungsten sheet, which may be easily cut to the desired shape, is found to be sufficiently dense, flexible, and uniform for clinical use as a skin collimation of small electron fields.

## CONFLICT OF INTEREST

The authors have no conflicts to disclose.

## AUTHOR CONTRIBUTIONS

Dylan Yamabe Breitkreutz, Lawrie Skinner, and Amy Yu contributed to the writing of the manuscript, measurements, analysis, and experimental design. Stephanie Lo contributed to experimental design and measurements.
